# Prospective evaluation of social risks, physical function, and cognitive function in prediction of non-elective rehospitalization and post-discharge mortality

**DOI:** 10.1186/s12913-022-07910-w

**Published:** 2022-04-29

**Authors:** Heather A. Clancy, Zheng Zhu, Nancy P. Gordon, Patricia Kipnis, Vincent X. Liu, Gabriel J. Escobar

**Affiliations:** 1 Systems Research Initiative, Kaiser Permanente Division of Research, 2000 Broadway Avenue, Oakland, CA 94612 USA; 2 Intensive Care Unit, Kaiser Permanente Medical Center, 700 Lawrence Expressway, Santa Clara, CA 95051 USA

**Keywords:** Social factors, Social determinants of health, Cognitive function, Physical function, Post-discharge outcomes, Readmission risk, Predictive modeling

## Abstract

**Background:**

Increasing evidence suggests that social factors and problems with physical and cognitive function may contribute to patients’ rehospitalization risk. Understanding a patient’s readmission risk may help healthcare providers develop tailored treatment and post-discharge care plans to reduce readmission and mortality. This study aimed to evaluate whether including patient-reported data on social factors; cognitive status; and physical function improves on a predictive model based on electronic health record (EHR) data alone.

**Methods:**

We conducted a prospective study of 1,547 hospitalized adult patients in 3 Kaiser Permanente Northern California hospitals. The main outcomes were non-elective rehospitalization or death within 30 days post-discharge. Exposures included patient-reported social factors and cognitive and physical function (obtained in a pre-discharge interview) and EHR–derived data for comorbidity burden, acute physiology, care directives, prior utilization, and hospital length of stay. We performed bivariate comparisons using Chi-square, t-tests, and Wilcoxon rank-sum tests and assessed correlations between continuous variables using Spearman’s rho statistic. For all models, the results reported were obtained after fivefold cross validation.

**Results:**

The 1,547 adult patients interviewed were younger (age, *p* = 0.03) and sicker (COPS2, *p* < 0.0001) than the rest of the hospitalized population. Of the 6 patient-reported social factors measured, 3 (not living with a spouse/partner, transportation difficulties, health or disability-related limitations in daily activities) were significantly associated (*p* < 0.05) with the main outcomes, while 3 (living situation concerns, problems with food availability, financial problems) were not. Patient-reported cognitive (*p* = 0.027) and physical function (*p* = 0.01) were significantly lower in patients with the main outcomes. None of the patient-reported variables, singly or in combination, improved predictive performance of a model that included acute physiology and longitudinal comorbidity burden (area under the receiver operator characteristic curve was 0.716 for both the EHR model and maximal performance of a random forest model including all predictors).

**Conclusions:**

In this insured population, incorporating patient-reported social factors and measures of cognitive and physical function did not improve performance of an EHR-based model predicting 30-day non-elective rehospitalization or mortality. While incorporating patient-reported social and functional status data did not improve ability to predict these outcomes, such data may still be important for improving patient outcomes.

**Supplementary Information:**

The online version contains supplementary material available at 10.1186/s12913-022-07910-w.

## Introduction

Increasing evidence suggests social factors like unstable housing [1]; food insecurity [2–7]; transportation difficulties [8]; chronic stress [9]; and ability to get help with activities of daily living [10–13] influence various health and healthcare utilization outcomes, including risk of hospitalization and frequent use of the emergency department (ED) [14]. Similarly, problems with physical and cognitive function may contribute to patients’ rehospitalization risk [15, 16]. Understanding a patient’s risk for readmission may help healthcare providers develop appropriate tailored treatment and post-discharge care plans to reduce potentially avoidable readmissions and mortality. Teasing out the independent contributions of these factors to health outcomes is complicated since they may coexist and correlate with illness or disability burden.

In 2015, we described the development of a real-time score – referred to as the Transitions Support Level (TSL) score – to predict rehospitalization and post-discharge mortality risk [17]. Subsequently, Kaiser Permanente Northern California (KPNC) hospitals instantiated this model in the electronic health record (EHR). The TSL employs longitudinal comorbidity, acute physiology, discharge care directives, and recent utilization [17] and generates a predicted percent risk for a composite outcome (non-elective hospitalization and/or death within 30 days following hospital discharge) ranging from 0 to 100%. Risk estimates based on the TSL were integrated with discharge and follow-up workflows, which has resulted in decreased rehospitalizations [18]. In that same year Kaiser Permanente’s (KP)’s Care Management Institute developed the Your Current Life Situation (YCLS) questionnaire and item bank to screen members for social factors that could affect health and healthcare access [19]. YCLS items have been used in a variety of pilot projects in different programs and settings but are not part of routine outpatient or inpatient assessment. During this time period, KPNC also started to evaluate the use of brief patient-reported measures of functional status.

To test the hypothesis that patient-reported information could enhance the TLS’s prediction of post-hospital outcomes over EHR data alone, we employed a prospective cohort study design. The aim of our study was to test the hypothesis that adding patient-reported data about social factors and physical and cognitive function to the TLS data would significantly enhance the ability to predict 30-day rehospitalization and mortality. A secondary aim was to determine what specific patient-reported information might be recommended for routine ascertainment and entry into the EHR.

## Materials and methods

### Setting

KPNC is an integrated healthcare delivery system that includes 21 hospitals and 257 medical offices [17, 20–24] serving ~ 4.5 million Kaiser Foundation Health Plan members in Northern California. The program completed deployment of the Epic EHR (www.epicsystems.com) in all its hospitals and clinics in mid-2010. For this study, we recruited patients at 3 KPNC hospitals (Oakland, San Leandro, Walnut Creek). The study was approved by the KPNC Institutional Review Board for the Protection of Human Subjects.

### Eligibility criteria

Patients were required to meet the following eligibility criteria: age ≥ 18 years; English speaker; insurance coverage other than Medicaid (Medi-Cal); inpatient or admitted for observation, other than Labor & Delivery service; current hospitalization began inside a KPNC hospital; discharge without further hospitalization elsewhere; not on infection isolation precautions; no “Comfort Care Only” order in effect at the time of discharge; and cognitively and functionally able to provide informed consent and answer questionnaires. The latter was determined in several ways: no diagnosis of dementia in EHR; ability to answer questions and provide informed consent without a proxy; and was alert, oriented, and approachable as verbally confirmed by the patient’s nurse or other hospital care provider. Since we were trying to interview patients as close to discharge date as possible, only patients with planned discharge that day or the following day were approached.

All hospitalized adults in KPNC are assigned an automated daily Transition Support Level (TSL) score every morning at 0600. The TSL score is based on a patient’s admission acute severity of illness, longitudinal comorbidity score, whether the patient experienced any hospitalizations in the 7 and 30 days preceding the index hospitalization, length of stay (truncated at 30 days), and discharge care directive (“full code” or not) [17]. Patients with a TSL of ≥ 25% on the day of discharge are automatically enrolled in the KPNC Transitions Program, and they receive additional in-person discharge services and extra follow-up calls.

We oversampled patients whose predicted risk was between 15–44% (low-middle range) for two reasons. First, based on published [17, 24] and internal analyses, we knew that most outcomes occurred above the 15% threshold (i.e., higher risk patients are more likely to experience adverse outcomes). Second, we reasoned that the greatest potential benefit would be to reclassify mid-level risk patients into either a higher or lower risk band, since very high-risk patients would be unlikely to have their risk estimates changed with new information and lower risk patients may not require additional intervention or follow-up.

### Recruitment and interview procedures (Fig. 1 and Appendix 2)

**Fig. 1:**
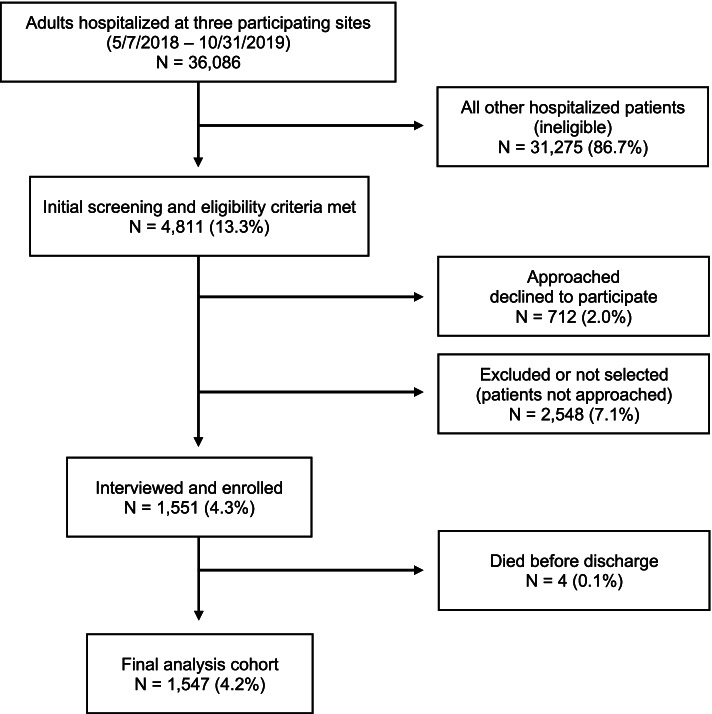
CONSORT diagram

Prior to recruitment, we obtained permission to approach patients from the study hospitals’ leadership and individual hospital attending physicians. Physician approvals permitted research staff to approach any patient who was under their care for the duration of the study, given that all eligibility and screening criteria were met. Research staff obtained confirmation from the patient’s nurse that the patient was alert, oriented, and approachable. Recruitment was conducted Monday-Friday between 9:00 AM and 5:00 PM from May 7, 2018 to October 31, 2019.

Research staff approached eligible patients and described the study. Patients who agreed to participate confirmed their identity using an authentication process, provided informed consent, signed an authorization form for use and disclosure of their information, and signed an acknowledgment of receipt of the KPNC Research Participants’ Bill of Rights. Patients then completed a staff-administered interview covering basic demographics, social risk factors, and physical and cognitive functioning. Staff used tablets to enter responses into a secure online data management tool separate from the EHR system, and data for the Patient Reported Outcomes Measurement Information System (PROMIS) questionnaires were entered directly into the PROMIS online Assessment Center (www.assessmentcenter.net). Participants received a $10 gift card/code as a token of our appreciation.

### Study measures

#### Research staff administered interview questionnaires

Our interview questionnaire included the National Institutes of Health’s PROMIS Physical Function and Cognitive Function Abilities Subset questionnaires [25] and social factors items derived from the KP YCLS item bank. PROMIS measures have a mean of 50 and a standard deviation (SD) of 10 in a referent population. Means *above* 50 and means within 0.5 SDs *below* 50 are considered within normal limits for function. For our final analyses, we created six social factor predictor variables based on the YCLS items: (1) relationship/marital status (married or living with partner); (2) food insecure (had worried about running out of food sometimes in the prior 3 months or anticipates having trouble paying for food in next 3 months); (3) housing-related concerns (in a temporary housing situation or homeless, concerned about housing conditions, or anticipates having trouble paying for housing or utilities in next 3 months); (4) financial strain (anticipates having trouble paying for ≥ 1 of 9 basic expenses in next 3 months); (5) transportation difficulties (anticipates problems with transportation during next 3 months); and (6) disability and help status (3-level variable: no health problem or disability that limits normal daily activities, limited but has ready access to help with medical needs and daily activities; limited but lacks ready access to help) (see Appendix 1: Interview Instruments).

### Electronic health record data

In addition to the EHR information needed to create the composite outcome (non-elective rehospitalization or 30-day mortality post-discharge), we confirmed that patients had Kaiser Foundation Health Plan membership, and extracted the TSL and its individual components (patients’ longitudinal comorbidity burden, severity of illness, length of stay, past healthcare utilization, and code status [17, 23]). As previously described [26], all adults with a KPNC medical record number are assigned a monthly COmorbidity Point Score, version 2 (COPS2, based on Centers for Medicare and Medicaid Services Hierarchical Condition Categories), with increasing COPS2 scores associated with increasing mortality risk [23]. Additionally, patients were assigned a Laboratory-based Acute Physiology Score, version 2 (LAPS2) [23] on admission and every hour after hospitalization. Increasing LAPS2 scores reflect worsening instability – for example, in July 2018, the median hourly LAPS2 among all KPNC intensive care unit patients was 110, whereas the median ward score was 52. It is not possible to admit a patient to KPNC hospitals without specifying code status, which can be subsequently updated. We classified each patient’s care directive as “full code” or “other” (which included “partial code,” “do not resuscitate,” and “comfort care only”) [23]. To compare, we assigned each hospitalization a Charlson Comorbidity Index score (CCI) using the methodology of Deyo et al. [27].

We extracted age at hospitalization; sex; self-reported race, hospitalization venue (via the ED or not); total index hospital length of stay (LOS); whether a patient experienced any overnight inpatient hospitalization in the first 7 days and separately in the 8 to 30 days preceding the index hospitalization [17]; discharge disposition (home; regular or custodial skilled nursing facility, SNF; and Home Health services); and referral to hospice. We classified the principal diagnosis codes using the Health Care Utilization Project (www.ahrq.gov/data/hcup) single-level diagnosis clinical classification software categories and clustered them into 30 groups called *Primary Conditions *[17, 23].

### Statistical methods

All analyses were done in SAS GRID, version 9.04.01M5P091317 and RStudio, version 1.3.1073. We performed bivariate comparisons using Chi-square, t-tests, and Wilcoxon rank-sum tests, as appropriate. We assessed correlations between continuous variables using Spearman’s rho statistic.

For our principal outcome analyses, we employed logistic regression. The dependent outcome was the same composite outcome (non-elective rehospitalization and/or death within 30 days of hospital discharge) that was used to calibrate the TSL score [17]. The independent variables included age, sex, the 6 above-mentioned social factors predictors, the 2 PROMIS scores, the aggregate TSL score, and all individual components of the TSL. In addition to testing various specific combinations (e.g., TSL + social factors items), we tested a random forest model that included all the individual above-mentioned predictors. We assessed model performance by measuring the area under the receiver operator characteristic curve (c statistic), Nagelkerke pseudo-R [2], and Brier score [28–30]. For all models, the results we report are those obtained after fivefold cross validation.

## Results

During the study recruitment period, 36,086 patients were hospitalized at the three participating sites. Of these patients, 4,811 met all initial screening and eligibility criteria as determined by the research staff. Of these, 2,263 patients were approached by research staff. Of these patients, 1,551 agreed to be interviewed and 712 declined to participate. The remaining 2,548 patients were excluded or not selected (i.e., not approached by research staff). See Fig. 1 for additional context.

During the recruitment period there were 36,086 adult patients hospitalized at the three Kaiser Permanente Northern California participating Medical Centers with an inpatient or observation designation. 2,548 patients not approached for various reasons were categorized as either “excluded” (*N* = 1,171) or “not selected” (*N* = 1,377). Excluded patients met most in-hospital eligibility criteria, but were not approached for another reason (i.e., nurse disapproved approaching patient, patient was already discharged, time constraints, or other as noted by research staff – such as “patient sleeping” or “family member visiting”). Patients “not selected” included those determined to be ineligible when research staff reviewed the patient’s electronic medical records on the day of recruitment. Not selected patients included: patients with discharge location unknown; patient continuing care at another facility after discharge; patient began their hospitalization at another non-KP facility and was transferred in; non-KP insurance coverage; comfort care only order in effect, could not confirm eligibility; or could not speak English. There were 1,551 patients interviewed and enrolled in the study. Four patients died prior to discharge and were removed from the analysis.

Table 1 shows the characteristics of patients during their selected hospitalization who were interviewed, excluded or not selected, and all other hospitalized patients (additional comparisons, Appendix 4).

**Table 1:** COHORT characteristics and unadjusted outcomes^a^

	**All other hospitalizations**	**Excluded or not selected**	**Interviewed and enrolled**	***P***
Number of patients^b^	31,275	2,548	1,551	–-
Age (Median, mean ± SD)	67.0, 64.4 ± 18.1	72.0, 69.6 ± 16.9	67.0, 65.2 ± 15.1	0.0333
Sex (% male)	48.9	48.5	46.5	0.0588
Race (%)				
White	52.6	55.8	54.7	0.0968
Black/African American	15.8	16.6	21.0	< .0001
Hispanic	14.0	11.4	9.1	< .0001
Asian	14.9	13.9	12.4	0.0044
Other/unknown race	2.6	2.4	2.7	0.8563
Charlson Comorbidity Index score^c^ (Median, mean ± SD)	2.0, 2.7 ± 2.8	3.0, 4.0 ± 3.2	3.0, 3.4 ± 3.0	< .0001
COPS2 (Median, mean ± SD)	21.0, 36.6 ± 38.3	45.0, 61.4 ± 56.0	31.0, 49.9 ± 47.2	< .0001
LAPS2 (Median, mean ± SD)	45.0, 53.1 ± 38.9	68.0, 70.2 ± 42.3	57.0, 59.5 ± 38.0	< .0001
Admitted for observation (%)	26.1	6.8	0.5	< .0001
Full code on admission (%)	89.8	80.5	92.3	0.0003
Ever admitted to ICU (%)	12.5	18.2	14.9	0.0103
Discharge diagnoses^d^ (%)				
Sepsis	12.1	18.8	19.5	< .0001
Community-acquired pneumonia	1.4	1.7	1.1	0.2546
Acute myocardial infarction	3.1	2.7	3.4	0.5818
Congestive heart fail	0.5	0.9	0.6	0.7275
Gastrointestinal bleeding	1.3	1.0	1.4	0.8527
All other	81.6	74.9	74.1	< .0001
Length of stay (days (Median, mean ± SD)	2.0, 3.4 ± 5.0	3.5, 5.9 ± 8.7	3.7, 5.0 ± 5.0	< .0001
Full code on discharge (%)	86.1	73.7	90.3	< .0001
TSL score^e^ (Median, mean ± SD)	9.0, 11.4 ± 7.9	12.0, 16.2 ± 11.7	9.0, 12.8 ± 9.5	< .0001
Died during initial hospitalization (%)	2.2	2.3	0.3	< .0001
Non-elective hospitalization within 30 days of discharge (%)	8.3	14.5	13.0	< .0001
Died within 30 days of discharge (%)	2.6	6.6	2.1	0.1878
Died or had non-elective hospitalization within 30 days of discharge (%)	10.3	19.6	13.6	0.0002

^a^Table 1 provides information on interviewed patients, patients who were excluded or not selected, and all remaining patients (except 712 patients who refused and whose data could not be used). See text, Figure 1, and Appendix 2 for additional details regarding the recruitment process. SD = standard deviation ICU = intensive care unit. The P value shown compares interviewed and enrolled patients to all other hospitalizations; additional comparisons are provided in Appendix 4

^b^During the study period, a total of 36,086 adult patients were hospitalized in Oakland, San Leandro, and Walnut Creek hospitals. Of these, 1,551 patients agreed to be interviewed, 4 of whom died prior to discharge resulting in 1547 patients in the final analysis cohort; 712 patients refused to participate, and we could not use their data; lastly, 2,548 patients were excluded or not selected. For comparison purposes, we selected the first hospitalization experienced by patients who had multiple hospitalizations during the study period (*N* = 31,275, far left column)

^c^The Charlson Comorbidity Index score (range, 0–40; higher scores indicate greater comorbidity burden) was calculated using the methodology of Deyo et al. [27]. COPS2 = COmorbidity Point Score, version 2 (COPS2, range, 0 to 1010, higher scores indicate increasing comorbidity burden) is assigned based on all diagnoses incurred by a patient in the 12 months preceding the index hospitalization. The univariate relationship of COPS2 with 30-day mortality is as follows: 0–39, 1.7%; 40–64, 5.2%; 65 + , 9.0%. LAPS2 = Laboratory-based Acute Physiology Score, version 2 (LAPS2, range, 0 to 414, higher scores indicating increasing physiologic derangement) is assigned based on a patient’s worst vital signs, pulse oximetry, neurological status, and 16 laboratory test results in the preceding 24 (hourly and discharge LAPS2) or 72 h (admission LAPS2). The univariate relationship of an admission LAPS2 with 30-day mortality is as follows: 0–59, 1.0%; 60–109, 5.0%; 110 + , 13.7%. See Escobar et al. [23]

^d^See text and Escobar et al. [23] for description of how we grouped diagnosis codes into Primary Conditions

^e^TSL = Transition Support Level score. This score is assigned at 6 AM on the day of discharge to all adult hospitalized patients in Kaiser Permanente Northern California. The score, which is expressed as a percent, is calibrated against a composite outcome (non-elective hospitalization and/or death within 30 days of discharge). It is based on a patient’s LAPS2, COPS2, length of stay, recent hospital and emergency department utilization preceding the current hospitalization, and discharge care directive (full code or not); see Escobar et al. [17] for details. Patients with a TSL score of ≥ 25% receive additional assessments and follow-up calls and appointments

The interviewed and enrolled cohort had more African American patients but fewer Hispanics. When compared to all other hospitalizations, interviewed patients were sicker, with higher CCI, COPS2, and LAPS2 scores. However, the interviewed cohort was healthier, with lower CCI, COPS2, and LAPS2 scores, than those who were excluded or not selected. Only 0.5% of the interviewed cohort was admitted for observation, and LOS was longer, but the distribution of discharge diagnoses was similar to that of the rest of the cohort. Four (0.3%) patients died in the hospital after their interview. In the remaining 1,547 interviewed patients, post-discharge mortality was somewhat lower than in the remaining patients. Compared to the rest of the hospitalized cohort, excluded patients’ TSL scores were higher (mean, 16.2, 11.4, *p* < 0.0001). Interviewed patients’ TSL scores were somewhat higher than the rest of the hospitalized cohort (12.8 vs. 11.4, *p* < 0.0001). Table 2 shows that, due to logistic difficulties, we were not successful in oversampling patients with TSL scores ≥ 15%. However, the table shows that the TSL score distribution of the interviewed patients is similar to that of all adult hospitalizations prior to the study and that weighting the sample was not necessary.

**Table 2:** Targeted and achieved sampling fractions^a^

**TSL SCORE STRATUM**^**b**^	**PRIOR TO STUDY**		**TARGETED DISTRIBUTION FOR STUDY**	**DISTRIBUTION ACHIEVED IN THE STUDY**	
	**% of all adult hospitalizations**	**Outcome rate among patients in this risk stratum**		**% of all interviewed hospitalized patients**	**Outcome rate among patients in this risk stratum**
< 15.0%	72%	~ 6%	36%	68.3%	7.5%
15.0–24.9%	16%	~ 18%	24%	17.2%	22.5%
25.0–44.9%	8%	~ 35%	23%	10.9%	30.9%
≥ 45%	4%	~ 50%	16%	3.6%	33.9%

^a^Table shows the distribution of patients in various risk strata prior to the study, the distribution we attempted to achieve, and the distribution we actually achieved.

^b^TSL = Transition Support Level score. The TSL is the percent risk for the study composite outcome (non-elective rehospitalization or death within 30 days of discharge). In this health system, patients with a score of ≥ 25% are automatically enrolled in the program’s follow-up protocols; patients with a score of ≥ 45% receive more intensive follow-up. See text and Escobar et al. [17] for details on this score.

Of the 1,547 interviewed patients who left the hospital alive, 212 (13.7%) experienced the adverse composite outcome. Table 3 shows that interviewed patients’ cognitive function (mean, 32.4) was outside the normalized PROMIS range, but their physical function (mean 53.6) was within the normalized PROMIS range.

**Table 3:** Relationship between predictors and composite outcome^a^

	**Interviewed patients in final cohort**	**Composite outcome present**	**Composite outcome absent**	***P***
	*N* = 1,547	*N* = 212	*N* = 1,335	
Age (Median, mean ± SD)	67.0, 65.2 ± 15.1	69.0, 68.0 ± 13.5	67.0, 64.8 ± 15.3	0.005
Sex (% Male)	46.5%	46.7%	46.5%	1.000
Charlson Comorbidity Index score^b^ (Median, mean ± SD)	3.0, 3.4 ± 3.0	5.0, 4.9 ± 3.3	3.0, 3.1 ± 2.9	< 0.001
COPS2^b^ (Median, mean ± SD)	31.0, 50.0 ± 47.3	72.0, 78.2 ± 55.4	28.0, 45.5 ± 44.3	< 0.001
LAPS2^b^ (Median, mean ± SD)	57.0, 59.4 ± 38.0	71.0, 74.4 ± 40.0	54.0, 57.0 ± 37.2	< 0.001
TSL^c^ (Median, mean ± SD)	10.8, 14.9 ± 11.4	18.6, 22.2 ± 15.1	10.3, 13.7 ± 10.2	< 0.001
Cognitive Function^d^ (Median, mean ± SD)	31.8, 32.4 ± 9.3	30.8, 30.8 ± 8.5	31.8, 32.6 ± 9.4	0.009
Physical Function^d^ (Median, mean ± SD)	53.8, 53.6 ± 10.0	52.3, 51.7 ± 10.2	53.9, 53.8 ± 9.9	0.005
YCLS^e^ items (%)				
Not married, not living with partner	47.1%	54.2%	45.9%	0.029
Housing difficulties present	15.1%	18.9%	14.5%	0.125
Food availability problems present	8.4%	8.5%	8.4%	1.000
Financial problems present	18.9%	21.7%	18.4%	0.300
Transportation difficulties present	15.8%	22.2%	14.8%	0.008
Disability present	51.3%	66.0%	49.0%	< 0.001
Help availability in context of presence of disability^f^				< 0.001
No disability, issue of help not applicable	48.7%	34.0%	51.0%	
Disability present, help is available	43.5%	55.7%	41.6%	
Disability present, help is availability uncertain	7.8%	10.4%	7.4%	

^a^Composite outcome = non-elective rehospitalization (hospitalization that began in the emergency department) and/or death within 30 days after hospital discharge

^b^The Charlson Comorbidity Index score (range, 0–40; higher scores indicate greater comorbidity burden) was calculated using the methodology of Deyo et al. [27]. COPS2 = COmorbidity Point Score, version 2 (COPS2, range, 0 to 10, higher scores indicate increasing comorbidity burden) is assigned based on all diagnoses incurred by a patient in the 12 months preceding the index hospitalization. The univariate relationship of COPS2 with 30-day mortality is as follows: 0–39, 1.7%; 40–64, 5.2%; 65 + , 9.0%. LAPS2 = Laboratory-based Acute Physiology Score, version 2 (LAPS2, range, 0 to 414, higher scores indicating increasing physiologic derangement) is assigned based on a patient’s worst vital signs, pulse oximetry, neurological status, and 16 laboratory test results in the preceding 24 (hourly and discharge LAPS2) or 72 h (admission LAPS2). The univariate relationship of an admission LAPS2 with 30-day mortality is as follows: 0–59, 1.0%; 60–109, 5.0%; 110 + , 13.7%. See Escobar et al. (2013)

^c^TSL = Transition Support Level score. This score is assigned at 6 AM on the day of discharge to all adult hospitalized patients in Kaiser Permanente Northern California. The score, which is expressed as a percent, is calibrated against a composite outcome (non-elective hospitalization and/or death within 30 days of discharge). It is based on a patient’s LAPS2, COPS2, length of stay, recent hospital and emergency department utilization preceding the current hospitalization, and discharge care directive (full code or not); see Escobar et al. [17] for details. Patients with a TSL score of ≥ 25% receive additional assessments and follow-up calls and appointments

^d^Patient Reported Outcomes Measurement Information System Cognitive Function bank v. 2.0 and Physical Function bank v. 2.0. There were 29 patients with missing Cognitive Function and 36 with missing Physical Function

^e^YCLS = Your Current Life Situation questionnaire. See Appendix 1 for details

^f^Among patients reporting the presence of a disability (*N* = 140/212 among patients with the composite outcome, 654/1,335 among those without), the proportions with help available were higher among those with the composite outcome (66.0% among those with the composite outcome, 49.0% among those without, *p* =  < .001)

Table 3 shows the two most common social risk factors as presence of a health problem or disability (51.3%) and not living with a partner (47.1%); the prevalence of the remaining social risk factors ranged from 8.4%-15.8%. Table 3 also compares the 212 patients who experienced the composite outcome with the 1335 patients who did not. Patients with the composite outcome had significantly higher CCI, COPS2, LAPS2, and TSL scores (all comparisons, *p* < 0.0001), and their cognitive function (30.8 vs. 31.8, *p* = 0.027) and physical function (52.3 vs. 53.9, *p* = 0.01) scores were lower than those who did not experience the outcome. Patients with the composite outcome were significantly more likely to have transportation difficulties (22.8% vs. 14.8%, *p* = 0.008) and to have a health problem or disability that affected their activities of daily living (66.0% vs. 49.0%, *p* < 0.001). Among patients with a health or disability issue, those with the composite outcome were more likely to report having help readily available.

Table 4 shows the performance of different multivariable models for predicting the composite outcome (Appendix 5 shows model coefficients). We found that incorporating the social factors and PROMIS variables did not improve model discrimination or explanatory power over the health and demographic data routinely captured in the EHR. Importantly, a random forest model that included all available predictors did not result in improved performance as compared to the logistic model with TSL. Analyses restricted to patients with mid-range (15.0–44.9%) TSL scores showed similar results.

**Table 4:** Performance of multivariable predictive models for composite outcome^a^

Model components	c	Nagelkerke pseudo-R^2^	Brier score
TSL^b^	0.716	0.066	0.112
TSL + age + sex	0.708	0.066	0.112
YCLS^c^	0.590	0.022	0.116
YCLS + age + sex	0.597	0.027	0.116
PROMIS scales^d^	0.555	0.010	0.117
PROMIS + age + sex	0.563	0.015	0.116
TSL + YCLS	0.695	0.074	0.111
TSL + PROMIS	0.703	0.068	0.111
TSL + YCLS + PROMIS	0.665	0.074	0.110
YCLS + PROMIS	0.536	0.025	0.186
Random forest^e^	0.715	0.060	0.112

^a^Models are calibrated against a composite outcome (non-elective rehospitalization – defined as a hospitalization that began in the emergency department – and/or death within 30 days after hospital discharge). c = c statistic, or area under receiver operator characteristic curve. All results are after fivefold cross validation

^b^TSL = Transition Support Level score. This score is assigned at 6 AM on the day of discharge to all adult hospitalized patients in Kaiser Permanente Northern California. The score, which is expressed as a percent, is calibrated against a composite outcome (non-elective hospitalization and/or death within 30 days of discharge). It is based on a patient’s LAPS2, COPS2, length of stay, recent hospital and emergency department utilization preceding the current hospitalization, and discharge care directive (full code or not); see Escobar et al. [17] for details. Patients with a TSL score of ≥ 25% receive additional assessments and follow-up calls and appointments

^c^YCLS = Your Current Life Situation questionnaire. See Appendix 1 for details

^d^Patient Reported Outcomes Measurement Information System Cognitive Function bank v. 2.0 and Physical Function bank v. 2.0. See Appendix 1 for details

^e^The random forest model included the following variables: age, sex, individual components of the TSL score, the two PROMIS scales, and the 6 YCLS components listed in Table 2

Correlations across continuous variables (age, LAPS2, COPS2, TSL, and PROMIS) and cross tabulations between these variables and individual social factor variables are provided in Appendix 3. Correlations between the 2 PROMIS scales and the other continuous variables were statistically significant but not necessarily clinically significant, with the highest correlation involving the PROMIS scales found for cognitive function and age (-0.15, *p* < 0.001). Age was positively correlated with severity of illness (LAPS2), comorbidity burden (COPS2), and TSL score. Cross tabulations between the continuous variables and individual social factors variables did not show a consistent pattern except with respect to the PROMIS physical function scale, where all patients with unfavorable social factors had significantly lower physical function (all 6 comparisons with *p* < 0.001).

## Discussion

Our prospective study with hospitalized patients found that adding patient-reported information about social factors, physical function, and cognitive status did not improve the statistical performance of a predictive model of rehospitalization or death within 30 days post-discharge compared to a model based on EHR clinical data alone. However, we did find that presence of unfavorable patient-reported social risks, lower physical function, and lower cognitive function were independently associated with a higher rate of the composite outcome.

Although we cannot compare our findings to other studies directly, our results are consistent with the findings of Bhavsar et al., who studied 8 outcomes (ED use, hospitalization, outpatient visit use, occurrence of myocardial infarction, stroke, asthma, accidents, and influenza). Their team found that adding neighborhood socioeconomic status variables did not improve upon the statistical performance of predictive models using routinely available clinical data from an EHR [31]. Our findings are also consistent with our own recent analyses, where we found that adding neighborhood socioeconomic status variables did not improve prediction models for mortality and 6 health care utilization variables [32].

It is important to highlight key limitations of our study. First, our cohort was restricted to an insured population in a health system with a relatively high degree of vertical outpatient-inpatient service integration. This integration is manifest in the availability of services aimed at preventing hospitalization, such as chronic condition management programs, a central call center, electronic patient portals, and automated risk detection, which have resulted in a steady decrease in rehospitalization and post-discharge mortality [24]. Second, due to resource limitations, we could not interview patients on weekends, nor could we interview non-English speakers, which latter effect is most evident in the reduced proportion of Hispanic patients in our cohort. Because of state of California regulatory requirements, we could not interview very low- income Kaiser Foundation Health Plan members covered through Medi-Cal (California’s Medicaid program). Thus, it is possible that, because of our setting and selection process, our study sample may not be generalizable to other settings. Third, since social factors and measures of physical and cognitive function were all patient-reported, it is possible that people’s responses did not accurately reflect their true level of vulnerability. These aforementioned exclusions and limitations may have resulted in an overall lower risk hospitalized cohort, thereby reducing our ability for our measures to impact the score. Fourth, it is possible that confounding or redundancy factors were occurring. For example, patients reporting health or disability problems who also had readily available help from others were more likely to experience the adverse composite outcome. This could be because people with health or disability problems that limit their ability to take care of their daily needs are more likely to have help availability than those not so limited. Lastly, our sample might consist of very similar patients for several reasons making it more difficult for a model to discriminate patients by their future outcome. The sample comes from a capitated staff model managed care insured population where patients at the extremes of the social and functional scales might not be represented. We also sampled only low-mid risk patients, and the sample of interviewed patients appeared to be healthier and lower risk than those excluded from the study.

It is important to consider, however, that the most important factor affecting our results is that the outcome time frame (30 days after discharge) is very narrow. Most comorbid conditions develop over many years, and it seems likely that by the time a patient has been hospitalized, the effect of social factors and lower physical and cognitive function has already manifested as a set of comorbid conditions. Not surprisingly then, predictive models that include quantification of comorbidities and acute physiology are unlikely to be affected by including factors that may have existed for years prior to the hospitalization and predisposing patients to develop conditions resulting in hospitalization.

The fact that the patient-reported social factors and PROMIS cognitive and physical function status variables we employed failed to enhance prediction for a 30-day outcome does not mean that ascertaining this information is not valuable. Having this information available in the EHR at time of discharge planning could be of significant value in designing tailored interventions and in assessing which patients might benefit most from specific interventions [33]. For example, patients with social risks or current social needs could be triaged to KPNC Continuum of Care programs such as Hospital-to-Home Transitions and Complex Needs Programs for follow-up in the outpatient setting. Additionally, it is likely that identifying and addressing social risks such as access to healthy food, transportation, a stable and supportive living situation, instrumental and emotional social support, and health literacy will have a positive impact on the recovery process, including reduction in medical complications and potentially preventable ED and outpatient visits. We have shown that, when information on benefit from an intervention is available, this information can be used to develop more accurate rehospitalization predictive models [34]. These variables could enhance the predictive ability of other outcomes in different populations, so it is important to continue to evaluate and quantify the value of collecting these data in studies such as this one.

It is important to recognize another key strength of this study. We were able to successfully collect social factors, physical function, and cognitive status patient-reported information during an inpatient stay using standardized questionnaires administered via tablet. It is feasible to collect this data and knowing this information about a patient at discharge may allow for more complex and tailored interventions aimed at improving outcomes and reducing readmissions [35].

## Supplementary Information

**Additional file 1: Appendix 1**. Interview instruments. **Appendix 2**. Expanded detail on sampling & recruitment. **Appendix 3**. Supplemental analyses in the interviewed population. **Appendix 4**. Table 1 expanded cohort characteristics and unadjusted outcomes. **Appendix 5**. Logistic regression beta coefficients and odds ratios for the predictive models for composite outcome.

## Data Availability

The datasets generated and/or analyzed during the current study are not publicly available due to their being the property of Kaiser Foundation Health Plan, Inc., but are available to interested collaborators in the context of a formal collaboration approved by the Kaiser Permanente Northern California Institutional Review Board for the Protection of Human Subjects. Dataset requests can be sent to either Dr. Patricia Kipnis, corresponding author, or Ms. Heather A. Clancy, first author.

## Abbreviations

CCI: Charlson Comorbidity Index score
COPS2: COmorbidity Point Score, version 2 (based on Centers for Medicare and Medicaid Services Hierarchical Condition Categories)
ED: Emergency department
EHR: Electronic health record
KPNC: Kaiser Permanente Northern California
LAPS2: Laboratory-based Acute Physiology Score, version 2
LOS: Length of stay
PROMIS: Patient Reported Outcomes Measurement Information System
SD: Standard deviation
TSL: Transition Support Level
YCLS: Your Current Life Situation
