# Supplementary material for: Prospective evaluation of social risks, physical function, and cognitive function in prediction of non-elective rehospitalization and post-discharge mortality
Source: BMC Health Serv Res. 2022 Apr 29;22:574. doi: 10.1186/s12913-022-07910-w (PMC9052530; doi:10.1186/s12913-022-07910-w)
Supplement: Supplementary file 1 — Additional file 1: Appendix 1. Interview instruments. Appendix 2. Expanded detail on sampling & recruitment. Appendix 3. Supplemental analyses in the interviewed population. Appendix 4. Table 1 expanded cohort characteristics and unadjusted outcomes. Appendix 5. Logistic regression beta coefficients and odds ratios for the predictive models for composite outcome. [file 12913_2022_7910_MOESM1_ESM.docx]

**PROSPECTIVE EVALUATION OF SOCIAL RISKS, PHYSICAL FUNCTION, AND COGNITIVE FUNCTION IN PREDICTION OF NON-ELECTIVE REHOSPITALIZATION AND POST-DISCHARGE MORTALITY**

Running title: *Prospective evaluation of patient-reported predictors for non-elective rehospitalization*

Heather A. Clancy, MPH; Zheng Zhu, MS; Nancy P. Gordon, ScD; Patricia Kipnis, PhD; Vincent X Liu, MD, MS; Gabriel J. Escobar, MD

**APPENDICES FOR INTERESTED READERS**

APPENDIX 1: INTERVIEW INSTRUMENTS

1-A YOUR CURRENT LIFE SITUATION (YCLS / SOCIAL FACTORS QUESTIONNAIRE) 2

1-B STRUCTURING SOCIAL FACTORS VARIABLES FOR ANALYSIS 3

APPENDIX 2: EXPANDED DETAIL ON SAMPLING & RECRUITMENT 4

APPENDIX 3: SUPPLEMENTAL ANALYSES IN THE INTERVIEWED POPULATION

3-A: CORRELATION MATRIX FOR CONTINUOUS PREDICTORS IN INTERVIEWED COHORT 5

3-B TO 3-H: CROSS TABULATIONS BETWEEN SOCIAL FACTORS ITEMS AND CONTINUOUS VARIABLES 5

APPENDIX 4: TABLE 1 EXPANDED COHORT CHARACTERISTICS AND UNADJUSTED OUTCOMES 8

APPENDIX 5: LOGISTIC REGRESSION BETA COEFFICIENTS AND ODDS RATIOS FOR THE PREDICTIVE MODELS FOR COMPOSITE OUTCOME 11

# **APPENDIX 1: INTERVIEW INSTRUMENTS**

## **1-A YOUR CURRENT LIFE SITUATION (YCLS / SOCIAL FACTORS QUESTIONNAIRE)**

1. What was the highest grade or level of school that you have completed? *(Select ONE only)*

| □ a. 8th grade or less  □ b. Some high school, but did not graduate  □ c. High school graduate or GED  □ d. Some college or Associates degree  □ e. Bachelor’s degree (B.A., B.S., etc.)  □ f. Graduate/professional degree (MA, PhD, MD, etc.) |
| --- |

2. Which of the following best describes your current living situation? *(Select ONE only)*

□ a. Live alone in my own home (house, apartment, condo, trailer, etc.); may have a pet

□ b. Live in a household with other people

□ c. Live in a residential facility where meals and household help are routinely provided by paid staff (or could be if requested)

□ d. Live in a facility such as a nursing home which provides meals and 24-hour nursing care

□ e. Temporarily staying with a relative or friend

□ f. Temporarily staying in a shelter or homeless

□ g. Other

3. What is your current marital/relationship status? *(Select ONE only)*

□ a. Married, domestic partner, or living with partner

□ b. In a committed relationship but not living together

□ c. Single, divorced, separated

□ d. Widowed

4. Do you have any concerns about your current living situation, like housing conditions, safety, and costs?

| \| □ Yes 🡪  □ No \| *Select ALL concerns that you have:*  □ a. Condition of housing  □ b. Lack of more permanent housing  □ c. Ability to pay for housing or utilities \| □ d. Feeling safe  □ e. Other \| \| --- \| --- \| --- \| |
| --- | --- | --- | --- |

5. In the past 3 months, how often have you worried that your food would run out before you had money to buy more?

□ Never □ Sometimes □ Often □ Very often

6. Over the next 3 months, do you think you will have trouble paying for any of the following? *(Select ALL that apply)*

| □ a. Food  □ b. Housing  □ c. Heat and electricity  □ d. Medical needs  □ e. Transportation | □ f. Childcare  □ g. Debts  □ h. Other  □ i. None of these |
| --- | --- |

7. How often do you need to have someone help you when you read instructions, pamphlets, or other written material from your doctor or pharmacy?

□ Never □ Rarely □ Sometimes □ Often □ Always

8. Over the next 3 months, do you think problems with transportation will:

a. Keep you from medical appointments? □ Yes □ No

b. Keep you from doing things needed for daily living? □ Yes □ No

9. If you need help with care related to your medical condition or with activities of daily living such as bathing, cooking, or shopping, are there people close by who will help you?

□ Yes □ No □ Not sure

10. Finally, do you have a health problem or disability that normally limits your activities or what you can do?

□ Yes □ No

**1-B STRUCTURING SOCIAL FACTORS VARIABLES FOR ANALYSIS**

The logic for converting the Social Factors components into the 6 predictors used in our analyses is provided below.

**MARITAL:** Not married, not living with partner

IF answer to 3 is “Single, divorced, separated” OR “Widowed” then MARITAL = 1

Else MARITAL = 0

**HOUSPROB:** Housing difficulties present

IF answer to 2 = “temporarily staying with a relative or friend” OR “temporarily staying in a shelter or homeless” OR “other”

OR if answer to 4 = “condition of housing” OR “lack of more permanent housing” OR “ability to pay for housing or utilities”

OR if answer to 6 = “housing” then HOUSPROB = 1

Else HOUSPROB = 0

**FOODPROB:** Food availability problems present

IF answer to 5 = “sometimes” OR “often” OR “very often”

OR if answer to 6 = “food” then FOODPROB = 1

Else FOODPROB = 0

**FINCPROB:** Financial problems present

IF answer to 6 = “none of these” then FINCPROBT = 0

Else FINCPROBT = ∑ (4c, 6a, 6b, 6c, 6d, 6e, 6f, 6g, 6h)

IF FINCPROBT ≥ 1, then FINCPROB = 1

Else FINCPROB = 0

**TRANPROB:** Transportation difficulties present

IF answer to 8 = “keep you from medical appointments” AND “keep you from doing things needed for daily living” is no, then TRANPROB = 0

Else TRANPROB = 1

**HELPSTAT:** Disability and availability of help given presence of a disability

| Answer to Social Factors question 9 (availability of help) | Answer to Social Factors question 10 (presence of limiting disability or problem | |
| --- | --- | --- |
|  | No | Yes |
| No | Group 1 (reference)  *No limiting disability, issue of help is not applicable* | Group 2 (worst)  *Limiting disability present, and not sure if help is available* |
| Not sure |  |  |
| Yes |  | Group 3 (intermediate) |

# **APPENDIX 2: EXPANDED DETAIL ON SAMPLING & RECRUITMENT**

To determine patient eligibility, research staff applied a multi-step screening and eligibility process. All criteria are listed below.

**STEP 1**

Patients had to meet all initial “electronic screening” criteria. These criteria were incorporated into the programming code that generated a list of potentially eligible patients.

- Patient is a KPNC member
- Patient is adult (18 years and older)
- Hospital status is inpatient or admitted for observation
- Hospitalization is not to the labor and delivery service
- Patient must be able to speak English

**STEP 2**

We then applied physician approval criteria to these patients. Prior to launching the study, we asked physicians from the 3 participating Sites whether they would agree to have research staff approach and interview patients under their care for the duration of the study. Patients whose physician gave their approval were moved to the next step and included on the “Daily Download” list.

- Physician Approval documented

**STEP 3**

The Daily Download list was generated every weekday morning and accessed by research staff to begin their recruitment process. Research staff confirmed criteria from Steps 1 and 2 and also applied additional screening criteria, which was done manually by reviewing the patient’s EMR. If information could not be readily confirmed in the patient’s EMR, research staff would confirm with the patient’s nurse or other care provider.

Additionally, research staff did not approach any patient whose hospital staff recommended not to approach for any reason. Patients who are unable to provide informed consent or answer questionnaires, are combative, confused, or on infection isolation precautions, or undergoing suicide prevention observation were not approached. As noted in the table below, research staff asked for a verbal confirmation from the patient’s nurse confirming that the patient was OK to approach.

- Confirm criteria from Steps 1 and 2
- Patient does not have a “Comfort Care Only” order in effect at the time of discharge
- Current hospitalization did not begin at a non-KPNC hospital (i.e., Patient is not eligible if they are a “transfer-in” from a non-KPNC hospital. Patient is eligible if they are a transfer-in from another KP hospital.)
- Patient is not being discharged directly to another hospital (i.e., Patient is not eligible if discharge is a “transfer-out” and hospitalization is continuing. We are including patients who are discharged to home, home health, assisted living facility, board and care, SNF, or custodial SNF. Patients being discharged to other locations (e.g., jail, another hospital, acute care facility, hospice, etc.) are not included.)
- Not Medi-Cal (NOTE: Medi-Cal patients were excluded due to regulatory constraints. To include Medi-Cal patients in research, it is required to obtain authorization from Medi-Cal first, which is inhibitive.)
- Patient is not on Infection Isolation Precautions (e.g., contact, protective, droplet, airborne, etc.)
- Patient is cognitively and functionally able to provide informed consent and answer questionnaires (i.e., Patient is not eligible if diagnosed with dementia, has other noted confusion or diminished cognitive capacity, or has difficulty hearing, speaking, or reading.)
- Patient is planned for discharge today or tomorrow
- Nurse gives verbal confirmation that patient is OK to approach

**STEP 4**

After all eligibility criteria was met, research staff made several additional checks prior to approaching the patient. These eligibility criteria are included as a separate step because they were not part of our original, pre-determined eligibility screening protocol. It is criteria that was developed as a result of our experience in the field and was also applied manually by research staff on the day that the patients were approached.

- For patients with multiple hospitalizations during the study period, patient has not been previously approached and recruited or approached and refused
- Break the Glass: The review of medical records is restricted when potential privacy can be compromised. The Break the Glass protocol enables KP providers to track who logs into the patient chart (e.g., when an employee is now a patient). The restriction placed on a patient’s EMR must be approved at a higher administrative level before it is applied.
- Patient is not under the “Green Blanket Protocol”. This protocol identifies patients that have proven to be or have the potential to be combative or violently impulsive. The green blanket tells anyone who enters the room to be careful.

# **APPENDIX 3: SUPPLEMENTAL ANALYSES IN THE INTERVIEWED POPULATION**

## **3-A: CORRELATION MATRIX FOR CONTINUOUS PREDICTORS IN INTERVIEWED COHORT**

|  | **Age** | **LAPS2** | **COPS2** | **TSL*** | **PROMIS – cognitive** | **PROMIS - physical** |
| --- | --- | --- | --- | --- | --- | --- |
| Age | --- | 0.16 (<.001) | 0.31 (<.001) | 0.26 (<.001) | -0.15 (<.001) | -0.04 (<0.1384) |
| LAPS2 | --- | --- | 0.30 (<.001) | 0.61 (<.001) | 0.06 (0.027) | -0.02 (0.340) |
| COPS2 | --- | --- | --- | 0.71 (<.001) | -0.10 (<.001) | -0.11 (<.001) |
| TSL | --- | --- | --- | --- | -0.09 (0.001) | -0.11 (<.001) |
| PROMIS – cognitive | --- | --- | --- | --- | --- | 0.21 (<.001) |
| PROMIS - physical | --- | --- | --- | --- | --- | --- |

***** TSL = Transition Support Level score.

## **3-B TO 3-H: CROSS TABULATIONS BETWEEN SOCIAL FACTORS ITEMS AND CONTINUOUS VARIABLES**

**TABLE 3-B: Not married, not living with partner**

| **Not married, not living with partner** | **Age** | **LAPS2** | **COPS2** | **TSL** | **PROMIS – cognitive** | **PROMIS - physical** |
| --- | --- | --- | --- | --- | --- | --- |
| Yes | 69.0 (58.8 -78.0) | 61.0 (30.0 -86.0) | 42.0 (13.0 -81.0) | 12.1 (8.0 -20.0) | 31.8 (23.9 -37.8) | 53.2 (45.4 -58.8) |
| No | 66.0 (56.0 -74.0) | 52.0 (16.0 -81.0) | 27.0 (10.0 -68.5) | 10.1 (7.1 -16.2) | 31.8 (23.7 -39.3) | 54.0 (47.6 -61.3) |
| p | <0.001 | 0.001 | <0.001 | <0.001 | 0.489 | 0.003 |

**TABLE 3-C: Housing difficulties present**

| **Housing difficulties present** | **Age** | **LAPS2** | **COPS2** | **TSL** | **PROMIS – cognitive** | **PROMIS - physical** |
| --- | --- | --- | --- | --- | --- | --- |
| Yes | 63.0 (53.0 -72.0) | 62.0 (24.5 -84.5) | 36.0 (10.0 -81.0) | 10.5 (7.2 -18.5) | 31.8 (23.9 -35.9) | 51.0 (44.4 -56.9) |
| No | 68.0 (58.0 -77.0) | 56.0 (22.0 -84.0) | 30.0 (10.0 -74.0) | 10.9 (7.4 -17.6) | 31.8 (23.9 -38.8) | 53.9 (47.4 -61.0) |
| p | <0.001 | 0.214 | 0.319 | 0.866 | 0.624 | <0.001 |

**TABLE 3-D: Food availability problems present**

| **Food availability problems present** | **Age** | **LAPS2** | **COPS2** | **TSL** | **PROMIS – cognitive** | **PROMIS - physical** |
| --- | --- | --- | --- | --- | --- | --- |
| Yes | 58.5 (47.3 -70.0) | 52.0 (19.0 -80.5) | 25.5 (10.0 -65.8) | 9.4 (6.8 -14.1) | 31.9 (23.9 -38.5) | 50.5 (43.4 -55.4) |
| No | 68.0 (58.0 -76.0) | 58.0 (24.0 -84.0) | 34.0 (10.0 -77.0) | 11.0 (7.5 -18.1) | 31.8 (23.9 -38.7) | 53.9 (47.0 -60.9) |
| p | <0.001 | 0.223 | 0.138 | 0.007 | 0.962 | <0.001 |

**TABLE 3-E: Financial problems present**

| **Financial problems present** | **Age** | **LAPS2** | **COPS2** | **TSL** | **PROMIS – cognitive** | **PROMIS - physical** |
| --- | --- | --- | --- | --- | --- | --- |
| Yes | 63.0 (51.0 -73.0) | 62.0 (27.0 -87.3) | 32.5 (10.0 -78.0) | 10.5 (7.4 -19.2) | 31.6 (23.7 -36.6) | 52.5 (44.0 -57.6) |
| No | 68.0 (58.0 -77.0) | 56.0 (22.0 -83.0) | 31.0 (10.0 -75.0) | 10.9 (7.4 -17.3) | 31.8 (23.9 -38.9) | 54.0 (47.2 -61.0) |
| p | <0.001 | 0.088 | 0.936 | 0.630 | 0.062 | <0.001 |

**TABLE 3-F: Transportation difficulties present**

| **Transportation difficulties present** | **Age** | **LAPS2** | **COPS2** | **TSL** | **PROMIS –cognitive** | **PROMIS - physical** |
| --- | --- | --- | --- | --- | --- | --- |
| Yes | 68.5 (60.0 -77.0) | 63.0 (23.8 -89.0) | 57.0 (21.0 -99.3) | 14.1 (8.5 -23.9) | 26.6 (23.2 -33.2) | 49.1 (42.7 -55.6) |
| No | 67.0 (56.0 -76.0) | 57.0 (22.0 -83.0) | 28.0 (10.0 -71.0) | 10.4 (7.3 -16.5) | 32.7 (24.5 -39.4) | 54.0 (47.6 -61.3) |
| p | 0.057 | 0.065 | <0.001 | <0.001 | <0.001 | <0.001 |

**TABLE 3-G: Presence of disability**

| **Disability present** | **Age** | **LAPS2** | **COPS2** | **TSL** | **PROMIS – cognitive** | **PROMIS - physical** |
| --- | --- | --- | --- | --- | --- | --- |
| Yes | 70.0 (59.3 -78.0) | 63.0 (33.3 -87.0) | 54.0 (21.0 -94.8) | 13.2 (8.7 -22.0) | 30.0 (23.7 -35.5) | 52.0 (44.5 -58.2) |
| No | 65.0 (54.0 -74.0) | 48.0 (16.0 -79.0) | 15.0 (10.0 -48.0) | 8.9 (6.7 -13.3) | 33.3 (25.8 -42.2) | 55.3 (49.2 -63.4) |
| p | <0.001 | <0.001 | <0.001 | <0.001 | <0.001 | <0.001 |

**TABLE 3-H: Presence of disability and availability of help given a disability**

| **Disability present, no help/help uncertain** | **Age** | **LAPS2** | **COPS2** | **TSL** | **PROMIS – cognitive** | **PROMIS - physical** |
| --- | --- | --- | --- | --- | --- | --- |
| Disability present, help availability uncertain | 70.0 (62.0 -78.0) | 60.0 (24.0-81.0) | 48.0 (21.0-83.0) | 13.2(8.3-19.5) | 26.7(23.6 -33.3) | 49.7 (44.3-56.7) |
| Disability present, help is available | 69.0 (59.0 -78.0) | 63.0 (35.0 -88.0) | 55.0 (21.0 -97.0) | 13.2 (8.9 -22.9) | 30.3 (23.7 -35.7) | 52.6 (44.6 -58.5) |
| No disability, issue of help not applicable | 65.0 (54.0 -74.0) | 48.0 (16.0 -79.0) | 15.0 (10.0 -48.0) | 8.9 (6.7 -13.3) | 33.3 (25.8 -42.2) | 55.3 (49.2 -63.4) |
| p | <0.001 | <0.001 | <0.001 | <0.001 | <0.001 | <0.001 |

| **APPENDIX 4: TABLE 1 EXPANDED COHORT CHARACTERISTICS AND UNADJUSTED OUTCOMES ^a^** | | | | | | | | |
| --- | --- | --- | --- | --- | --- | --- | --- | --- |
|  | **All other hospitalizations**  **(A)** | **Not selected**  **(B)** | **Excluded**  **(C)** | **Interviewed**  **(D)** | **P Value**  **D vs A** | **P Value**  **D vs B** | **P Value**  **D vs C** | **P Value**  **D vs A+B+C** |
| Number of patients ^b^ | 31,275 | 1,377 | 1,171 | 1,551 | --- | --- | --- | --- |
| Age (Median, mean ± SD) | 67.0, 64.4 ± 18.1 | 69.0, 66.9 ± 16.9 | 75.0, 72.9 ± 16.4 | 67.0, 65.2 ± 15.1 | 0.0333 | 0.0048 | <.0001 | 0.2562 |
| Sex (% male) | 48.9 | 49.3 | 47.6 | 46.5 | 0.0588 | 0.1269 | 0.5763 | 0.0618 |
| Race (%) | | | | | | | | |
| White | 52.6 | 51.9 | 60.4 | 54.7 | 0.0968 | 0.1277 | 0.0032 | 0.1398 |
| Black/African American | 15.8 | 16.0 | 17.2 | 21.0 | <.0001 | 0.0005 | 0.0109 | <.0001 |
| Hispanic | 14.0 | 12.7 | 9.8 | 9.1 | <.0001 | 0.0018 | 0.5206 | <.0001 |
| Asian | 14.9 | 16.1 | 11.3 | 12.4 | 0.0044 | 0.0046 | 0.3481 | 0.0057 |
| Other/unknown race | 2.6 | 3.2 | 1.4 | 2.7 | 0.8563 | 0.4380 | 0.0121 | 0.8174 |
| Charlson Comorbidity Index score ^c^ (Median, mean ± SD) | 2.0, 2.7 ± 2.8 | 3.0, 3.9 ± 3.3 | 4.0, 4.0 ± 3.0 | 3.0, 3.4 ± 3.0 | <.0001 | <.0001 | <.0001 | <.0001 |
| COPS2 (Median, mean ± SD) | 21.0, 36.6 ± 38.3 | 42.0, 60.6 ± 58.6 | 49.0, 62.2 ± 52.7 | 31.0, 49.9 ± 47.2 | <.0001 | <.0001 | <.0001 | <.0001 |
| LAPS2 (Median, mean ± SD) | 45.0, 53.1 ± 38.9 | 62.0, 66.7 ± 43.1 | 73.0, 74.4 ± 41.0 | 57.0, 59.5 ± 38.0 | <.0001 | <.0001 | <.0001 | <.0001 |
| Admitted for observation (%) | 26.1 | 7.0 | 6.6 | 0.5 | <.0001 | <.0001 | <.0001 | <.0001 |
| Full code on admission (%) | 89.8 | 82.3 | 78.3 | 92.3 | 0.0003 | <.0001 | <.0001 | <.0001 |
| Ever admitted to ICU (%) | 12.5 | 19.2 | 17.2 | 14.9 | 0.0103 | 0.0022 | 0.1113 | 0.0355 |
| Discharge diagnoses ^d^ (%) | | | | | | | | |
| Sepsis | 12.1 | 17.7 | 20.2 | 19.5 | <.0001 | 0.2074 | 0.6889 | <.0001 |
| Community-acquired pneumonia | 1.4 | 1.2 | 2.2 | 1.1 | 0.2546 | 0.7280 | 0.0262 | 0.2228 |
| Acute myocardial infarction | 3.1 | 3.3 | 2.0 | 3.4 | 0.5818 | 0.9855 | 0.0232 | 0.5391 |
| Congestive heart fail | 0.5 | 0.8 | 0.9 | 0.6 | 0.7275 | 0.4779 | 0.2934 | 0.8305 |
| Gastrointestinal bleeding | 1.3 | 1.2 | 0.8 | 1.4 | 0.8527 | 0.7752 | 0.1325 | 0.7981 |
| All other | 81.6 | 75.7 | 74.0 | 74.1 | <.0001 | 0.3218 | 0.9402 | <.0001 |
| Length of stay - # days (Median, mean ± SD) | 2.0, 3.4 ± 5.0 | 3.3, 5.5 ± 8.9 | 3.7, 6.3 ± 8.5 | 3.7, 5.0 ± 5.0 | <.0001 | 0.0745 | <.0001 | <.0001 |
| Full code on discharge (%) | 86.1 | 75.7 | 71.3 | 90.3 | <.0001 | <.0001 | <.0001 | <.0001 |
| TSL score^e^ (Median, mean ± SD) | 9.0, 11.4 ± 7.9 | 12.0, 16.4 ± 13.0 | 14.0, 16.0 ± 9.9 | 9.0, 12.8 ± 9.5 | <.0001 | <.0001 | <.0001 | <.0001 |
| Died during initial hospitalization (%) | 2.2 | 3.4 | 0.9 | 0.3 | <.0001 | <.0001 | 0.0281 | <.0001 |
| Non-elective hospitalization within 30 days of discharge | 8.3 | 3.9 | 27.0 | 13.0 | <.0001 | <.0001 | <.0001 | <.0001 |
| Died within 30 days of discharge | 2.6 | 7.5 | 5.6 | 2.1 | 0.1878 | <.0001 | <.0001 | 0.0345 |
| Died or had non-elective hospitalization within 30 days of discharge | 10.3 | 10.7 | 30.1 | 13.6 | 0.0002 | 0.0180 | <.0001 | 0.0032 |

**FOOTNOTES**

1. Table 1 Expanded Cohort provides information on interviewed patients, patients who were excluded, patients who were not selected, and all remaining patients (except 712 patients who refused and whose data could not be used). Excluded patients were those who met most in-hospital eligibility criteria, but were not approached for another reason (i.e., nurse disapproved approaching patient, patient was already discharged, time constraints, or another reason noted by research staff – some examples of notes included “patient sleeping” or “family member visiting”). Those patients who were “not selected” included those who were determined to be ineligible when research staff reviewed the patient’s EMR on the day of recruitment. Not selected patients included: patients with discharge location unknown; patient continuing care at another facility after discharge; patient began their hospitalization at another non-KP facility and was transferred in; non-KP insurance coverage; comfort care only order in effect, could not confirm eligibility; or could not speak English). See text, Figure 1, and Appendix 2 for additional detail regarding the recruitment process. IQR = interquartile range; ICU = intensive care unit. The P value shown compares interviewed and enrolled patients to all other hospitalizations.
2. During the study period, there were 36,086 adult patients hospitalized at the three KP Northern California (KPNC) participating Medical Centers with an inpatient or observation designation. Of these, there were 1,551 patients who agreed to be interviewed; 712 patients refused to participate in the study and we could not use their data, 1,377 patients were not selected; lastly, 1,171 patients were excluded.
3. The Charlson Comorbidity Index score (range, 0-40; higher scores indicate greater comorbidity burden) was calculated using the methodology of Deyo et al. (1992). COPS2 = COmorbidity Point Score, version 2 (COPS2, range, 0 to 1010, higher scores indicate increasing comorbidity burden) is assigned based on all diagnoses incurred by a patient in the 12 months preceding the index hospitalization. The univariate relationship of COPS2 with 30-day mortality is as follows: 0-39, 1.7%; 40-64, 5.2%; 65+, 9.0%. LAPS2 = Laboratory-based Acute Physiology Score, version 2 (LAPS2, range, 0 to 414, higher scores indicating increasing physiologic derangement) is assigned based on a patient’s worst vital signs, pulse oximetry, neurological status, and 16 laboratory test results in the preceding 24 (hourly and discharge LAPS2) or 72 hours (admission LAPS2). The univariate relationship of an admission LAPS2 with 30-day mortality is as follows: 0-59, 1.0%; 60-109, 5.0%; 110+, 13.7%. See Escobar et al. (2013).
4. See text and Escobar et al. (2013) for description of how we grouped diagnosis codes into Primary Conditions
5. TSL = Transition Support Level score. This score is assigned at 6 AM on the day of discharge to all adult hospitalized patients in Kaiser Permanente Northern California. The score, which is expressed as a percent, is calibrated against a composite outcome (non-elective hospitalization and/or death within 30 days of discharge). It is based on a patient’s LAPS2, COPS2, length of stay, recent hospital and emergency department utilization preceding the current hospitalization, and discharge care directive (full code or not); see Escobar et al. (2015) for details. Patients with a TSL score of ≥ 25% receive additional assessments and follow-up calls and appointments.

## **APPENDIX 5: LOGISTIC REGRESSION BETA COEFFICIENTS AND ODDS RATIOS FOR THE PREDICTIVE MODELS FOR COMPOSITE OUTCOME**

| **5-A. TSL^a^** |  |  |  |  |  |
| --- | --- | --- | --- | --- | --- |
| **Parameter** | **Beta Coefficient** | **Odd Ratio** | **SE** | **z-value** | **P(>\|z\|)** |
| Intercept | -2.684 | 0.07 | 0.129 | -20.818 | 0 |
| TSL | 0.049 | 1.05 | 0.005 | 9.031 | 0 |

**FOOTNOTE**

1. TSL = Transition Support Level score. This score is assigned at 6 AM on the day of discharge to all adult hospitalized patients in Kaiser Permanente Northern California. The score, which is expressed as a percent, is calibrated against a composite outcome (non-elective hospitalization and/or death within 30 days of discharge). It is based on a patient’s LAPS2, COPS2, length of stay, recent hospital and emergency department utilization preceding the current hospitalization, and discharge care directive (full code or not); see Escobar et al. (2015) for details. Patients with a TSL score of ≥ 25% receive additional assessments and follow-up calls and appointments.

|  |  |  |  |  |  |
| --- | --- | --- | --- | --- | --- |
| **5-B. TSL + Age + Sex** |  |  |  |  |  |
| **Parameter** | **Beta Coefficient** | **Odd Ratio** | **SE** | **z-value** | **P(>\|z\|)** |
| Intercept | -2.903 | 0.05 | 0.381 | -7.619 | 0 |
| TSL | 0.048 | 1.05 | 0.006 | 8.528 | 0 |
| Age | 0.004 | 1.00 | 0.006 | 0.639 | 0.523 |
| Male | 0.002 | 1.00 | 0.155 | 0.014 | 0.989 |
|  |  |  |  |  |  |
| **5-C. YCLS^a^** |  |  |  |  |  |
| **Parameter** | **Beta Coefficient** | **Odd Ratio** | **SE** | **z-value** | **P(>\|z\|)** |
| Intercept | -2.37 | 0.09 | 0.143 | -16.558 | 0 |
| Housing Difficulties | 0.198 | 1.22 | 0.22 | 0.902 | 0.367 |
| Food Availability Problems | -0.27 | 0.76 | 0.299 | -0.903 | 0.367 |
| Financial Problems | 0.021 | 1.02 | 0.21 | 0.099 | 0.921 |
| Transportation Difficulties | 0.26 | 1.30 | 0.199 | 1.306 | 0.191 |
| Limiting Disability and Uncertain Help | 0.556 | 1.74 | 0.287 | 1.939 | 0.053 |
| Disability | 0.626 | 1.87 | 0.164 | 3.814 | 0.0001 |
| Single, Divorced, Separated or Widowed | 0.227 | 1.25 | 0.153 | 1.48 | 0.139 |
|  |  |  |  |  |  |

**FOOTNOTE**

1. **YCLS** = Your Current Life Situation

| 5-D. YCLS + Age + Sex |  |  |  |  |  |
| --- | --- | --- | --- | --- | --- |
| **Parameter** | **Beta Coefficient** | **Odd Ratio** | **SE** | **z-value** | **P(>\|z\|)** |
| Intercept | -3.199 | 0.04 | 0.399 | -8.008 | 0 |
| Housing Difficulties | 0.222 | 1.25 | 0.221 | 1.006 | 0.314 |
| Food Availability Problems | -0.198 | 0.82 | 0.302 | -0.656 | 0.512 |
| Financial Problems | 0.07 | 1.07 | 0.212 | 0.329 | 0.742 |
| Transportation Difficulties | 0.26 | 1.30 | 0.2 | 1.301 | 0.193 |
| Limiting Disability and Uncertain Help | 0.479 | 1.61 | 0.289 | 1.658 | 0.097 |
| Disability | 0.577 | 1.78 | 0.166 | 3.476 | 0.001 |
| Single, Divorced, Separated or Widowed | 0.222 | 1.25 | 0.159 | 1.396 | 0.163 |
| Age | 0.012 | 1.01 | 0.005 | 2.128 | 0.033 |
| Male | 0.15 | 1.16 | 0.156 | 0.96 | 0.337 |

| **5-E. PROMIS** |  |  |  |  |  |  |  |  |  |  |  |
| --- | --- | --- | --- | --- | --- | --- | --- | --- | --- | --- | --- |
| **Parameter** | **Beta Coefficient** | **Odd Ratio** | **SE** | **z-value** | **P(>\|z\|)** |  |  |  |  |  |  |
| Intercept | -0.341 | 0.71 | 0.444 | -0.77 | 0.442 |  |  |  |  |  |  |
| PROMIS Cognitive | -0.018 | 0.98 | 0.009 | -2.049 | 0.04 |  |  |  |  |  |  |
| PROMIS Physical | -0.018 | 0.98 | 0.008 | -2.32 | 0.02 |  |  |  |  |  |  |
|  |  |  |  |  |  |  |  |  |  |  |  |
| **5-F. PROMIS + Age + Sex** |  |  |  |  |  |  |  |  |  |  |  |
| **Parameter** | **Beta Coefficient** | **Odd Ratio** | **SE** | **z-value** | **P(>\|z\|)** |  |  |  |  |  |  |
| Intercept | -1.268 | 0.28 | 0.593 | -2.138 | 0.032 |  |  |  |  |  |  |
| PROMIS Cognitive | -0.017 | 0.98 | 0.009 | -1.857 | 0.063 |  |  |  |  |  |  |
| PROMIS Physical | -0.018 | 0.98 | 0.008 | -2.337 | 0.019 |  |  |  |  |  |  |
| Age | 0.013 | 1.01 | 0.005 | 2.371 | 0.018 |  |  |  |  |  |  |
| Male | 0.132 | 1.14 | 0.156 | 0.85 | 0.396 |  |  |  |  |  |  |
|  |  |  |  |  |  |  |  |  |  |  |  |
| **5-G. TSL + YCLS** | | | | | |  |  |  |  |  |  |
| **Parameter** | **Beta Coefficient** | **Odd Ratio** | **SE** | **z-value** | **P(>\|z\|)** |  |  |  |  |  |  |
| Intercept | -2.982 | 0.05 | 0.17 | -17.503 | 0 |  |  |  |  |  |  |
| TSL | 0.046 | 1.05 | 0.006 | 8.019 | 0 |  |  |  |  |  |  |
| Housing Difficulties | 0.145 | 1.16 | 0.227 | 0.637 | 0.524 |  |  |  |  |  |  |
| Food Availability Problems | -0.03 | 0.97 | 0.307 | -0.097 | 0.922 |  |  |  |  |  |  |
| Financial Problems | -0.005 | 1.00 | 0.218 | -0.024 | 0.981 |  |  |  |  |  |  |
| Transportation Difficulties | 0.047 | 1.05 | 0.209 | 0.225 | 0.822 |  |  |  |  |  |  |
| Limiting Disability and Uncertain Help | 0.485 | 1.62 | 0.291 | 1.664 | 0.096 |  |  |  |  |  |  |
| Disability | 0.37 | 1.45 | 0.171 | 2.163 | 0.031 |  |  |  |  |  |  |
| Single, Divorced, Separated or Widowed | 0.211 | 1.23 | 0.157 | 1.343 | 0.179 |  |  |  |  |  |  |
|  |  |  |  |  |  |  |  |  |  |  |  |
| **5-H. TSL + PROMIS** |  |  |  |  |  |  |  |  |  |  |  |
| **Parameter** | **Beta Coefficient** | **Odd Ratio** | **SE** | **z-value** | **P(>\|z\|)** |  |  |  |  |  |  |
| Intercept | -1.649 | 0.19 | 0.491 | -3.359 | 0.001 |  |  |  |  |  |  |
| PROMIS Cognitive | -0.013 | 0.99 | 0.009 | -1.397 | 0.162 |  |  |  |  |  |  |
| PROMIS Physical | -0.011 | 0.99 | 0.008 | -1.409 | 0.159 |  |  |  |  |  |  |
| TSL | 0.047 | 1.05 | 0.006 | 8.375 | 0 |  |  |  |  |  |  |
|  |  |  |  |  |  |  |  |  |  |  |  |
| **5-I. TSL + YCLS + PROMIS** |  |  |  |  |  |  |  |  |  |  |  |
| **Parameter** | **Beta Coefficient** | **Odd Ratio** | **SE** | **z-value** | **P(>\|z\|)** |  |  |  |  |  |  |
| Intercept | -2.15 | 0.12 | 0.543 | -3.961 | 0.0001 |  |  |  |  |  |  |
| Housing Difficulties | 0.155 | 1.17 | 0.232 | 0.668 | 0.504 |  |  |  |  |  |  |
| Food Availability Problems | 0.021 | 1.02 | 0.312 | 0.069 | 0.945 |  |  |  |  |  |  |
| Financial Problems | -0.021 | 0.98 | 0.223 | -0.096 | 0.924 |  |  |  |  |  |  |
| Transportation Difficulties | -0.012 | 0.99 | 0.215 | -0.054 | 0.957 |  |  |  |  |  |  |
| Limiting Disability and Uncertain Help | 0.402 | 1.49 | 0.297 | 1.353 | 0.176 |  |  |  |  |  |  |
| Disability | 0.275 | 1.32 | 0.177 | 1.552 | 0.121 |  |  |  |  |  |  |
| Single, Divorced, Separated or Widowed | 0.2 | 1.22 | 0.159 | 1.255 | 0.209 |  |  |  |  |  |  |
| PROMIS Cognitive | -0.009 | 0.99 | 0.009 | -0.969 | 0.333 |  |  |  |  |  |  |
| PROMIS Physical | -0.009 | 0.99 | 0.008 | -1.069 | 0.285 |  |  |  |  |  |  |
| TSL | 0.045 | 1.05 | 0.006 | 7.712 | 0 |  |  |  |  |  |  |
|  |  |  |  |  |  |  |  |  |  |  |  |
| **5-J. YCLS + PROMIS** |  |  |  |  |  |  |  |  |  |  |  |
| **Parameter** | **Beta Coefficient** | **Odd Ratio** | **SE** | **z-value** | **P(>\|z\|)** |  |  |  |  |  |  |
| Intercept | -1.27 | 0.28 | 0.511 | -2.483 | 0.013 |  |  |  |  |  |  |
| Housing Difficulties | 0.188 | 1.21 | 0.224 | 0.839 | 0.401 |  |  |  |  |  |  |
| Food Availability Problems | -0.216 | 0.81 | 0.303 | -0.713 | 0.476 |  |  |  |  |  |  |
| Financial Problems | 0.015 | 1.02 | 0.215 | 0.068 | 0.946 |  |  |  |  |  |  |
| Transportation Difficulties | 0.164 | 1.18 | 0.205 | 0.797 | 0.425 |  |  |  |  |  |  |
| Limiting Disability and Uncertain Help | 0.48 | 1.62 | 0.292 | 1.645 | 0.1 |  |  |  |  |  |  |
| Disability | 0.528 | 1.70 | 0.169 | 3.119 | 0.002 |  |  |  |  |  |  |
| Single, Divorced, Separated or Widowed | 0.213 | 1.24 | 0.156 | 1.37 | 0.171 |  |  |  |  |  |  |
| PROMIS Cognitive | -0.011 | 0.99 | 0.009 | -1.213 | 0.225 |  |  |  |  |  |  |
| PROMIS Physical | -0.013 | 0.99 | 0.008 | -1.624 | 0.104 |  |  |  |  |  |  |
